# Polysaccharide of Ganoderma lucidum Ameliorates Cachectic Myopathy Induced by the Combination Cisplatin plus Docetaxel in Mice

**DOI:** 10.1128/spectrum.03130-22

**Published:** 2023-05-22

**Authors:** Sung-Yu Wu, Chu-Chyn Ou, Meng-Lin Lee, I-Lun Hsin, Yu-Ting Kang, Ming-Shiou Jan, Jiunn-Liang Ko

**Affiliations:** a Institute of Medicine, Chung Shan Medical University, Taichung, Taiwan; b Department of Ophthalmology, Chung Shan Medical University Hospital, Taichung, Taiwan; c Department of Nutrition, Chung Shan Medical University, Taichung, Taiwan; d Institute of Biochemistry, Microbiology and Immunology, Chung Shan Medical University, Taichung, Taiwan; e Department of Health Industry Technology Management, Chung Shan Medical University, Taichung, Taiwan; f Department of Medical Oncology and Chest Medicine, Chung Shan Medical University Hospital, Taichung, Taiwan; Tainan Hospital, Ministry of Health and Welfare

**Keywords:** chemotherapy, cachexia, side effect, *Ganoderma lucidum* polysaccharide, microbiota

## Abstract

Cachexia is a lethal muscle-wasting syndrome associated with cancer and chemotherapy use. Mounting evidence suggests a correlation between cachexia and intestinal microbiota, but there is presently no effective treatment for cachexia. Whether the Ganoderma lucidum polysaccharide Liz-H exerts protective effects on cachexia and gut microbiota dysbiosis induced by the combination cisplatin plus docetaxel (cisplatin + docetaxel) was investigated. C57BL/6J mice were intraperitoneally injected with cisplatin + docetaxel, with or without oral administration of Liz-H. Body weight, food consumption, complete blood count, blood biochemistry, and muscle atrophy were measured. Next-generation sequencing was also performed to investigate changes to gut microbial ecology. Liz-H administration alleviated the cisplatin + docetaxel-induced weight loss, muscle atrophy, and neutropenia. Furthermore, upregulation of muscle protein degradation-related genes (*MuRF-1* and *Atrogin-1*) and decline of myogenic factors (MyoD and myogenin) after treatment of cisplatin and docetaxel were prevented by Liz-H. Cisplatin and docetaxel treatment resulted in reducing comparative abundances of *Ruminococcaceae* and *Bacteroides*, but Liz-H treatment restored these to normal levels. This study indicates that Liz-H is a good chemoprotective reagent for cisplatin + docetaxel-induced cachexia.

**IMPORTANCE** Cachexia is a multifactorial syndrome driven by metabolic dysregulation, anorexia, systemic inflammation, and insulin resistance. Approximately 80% of patients with advanced cancer have cachexia, and cachexia is the cause of death in 30% of cancer patients. Nutritional supplementation has not been shown to reverse cachexia progression. Thus, developing strategies to prevent and/or reverse cachexia is urgent. Polysaccharide is a major biologically active compound in the fungus Ganoderma lucidum. This study is the first to report that *G. lucidum* polysaccharides could alleviate chemotherapy-induced cachexia via reducing expression of genes that are known to drive muscle wasting, such as *MuRF-1* and *Atrogin-1.* These results suggest that Liz-H is an effective treatment for cisplatin + docetaxel-induced cachexia.

## INTRODUCTION

Cachexia is a multifactorial syndrome driven by metabolic dysregulation, anorexia, systemic inflammation and insulin resistance. Cachexia is defined as 5% body weight loss within 12 months or 2% body weight loss with a body mass index (BMI) less than 20 (1, 2). Approximately 80% of advanced cancer patients have cachexia, and cachexia is the cause of death in 30% of cancer patients (3, 4). No effective treatment is currently available for cachexia, and nutritional supplementation has not been shown to reverse cachexia progression in cancer patients (5). Thus, developing treatment strategies for cachexia is urgent. At present, many studies have pointed out a strong correlation between cachexia and the intestinal microbiota (6–8). Modulating the intestinal microbiota may provide a strategy for treating this syndrome.

Chemotherapy is a primary treatment option for cancers. Since the mechanism of chemotherapy is widely killing proliferating cells, it also damages healthy cells, thus causing side effects such as neutropenia, gastrointestinal mucositis, and hepatotoxicity. Recent studies showed that chemotherapeutic agents alone are sufficient to induce cachexia occurrence and progression (9–13). Healthy rats demonstrated weight loss, anorexia, and negative nitrogen balance after receiving a single-dose intraperitoneal (i.p.) injection of chemotherapeutic drugs such as cyclophosphamide, 5-fluorouracil, cisplatin, and methotrexate (14). The mechanism of cisplatin-induced muscle atrophy is through upregulation of the NF-kappa B pathway (12) and activation of myostatin and the ubiquitin proteasome system in muscle cells (10, 15).

Many studies showed that chemotherapy reagents could significantly change the population of the gut microbiota (16, 17). Dysbiosis of the gut microbiota is also considered to be involved in the pathogenesis of cachexia. For example, after restoring *Lactobacillus* levels in mice with acute leukemia, the expression of atrophy markers *MurF-1* and *Atrogin-1* was inhibited in muscle tissue (18). Fecal microbiota transplantation (FMT) attenuated muscle wasting induced by 5-fluorouracil (19). Together, these results show that chemotherapy-induced cachexia might be through gut dysbiosis and that manipulating the gut microbiota is a potential treatment for chemotherapy-induced cachexia.

Ganoderma lucidum is a type of mushroom that has been used as a traditional medicine for centuries, and it has been found to have many potential health benefits. *G. lucidum* polysaccharide is a major biologically active compound in *G. lucidum*, and it has multiple functions, such as anticancer, hepatoprotection, anti-inflammatory, and antioxidation functions (20). Based on its 1,3- or 1,6-β-d-glucan chemical structure, *G. lucidum* polysaccharide cannot be digested by host-derived enzymes in the human gut. However, increasing evidence suggests that various nondigestible bioactive natural products, such as dietary fibers or polysaccharide, could be further converted into functional metabolites by the gut microbiota (21), meaning that although polysaccharides cannot be digested by human enzymes, they could still be decomposed into small molecules by bacteria.

*G. lucidum* polysaccharide was recently found to improve the anticancer effect of cisplatin and reduce the renal toxicity and hepatotoxicity of cisplatin (22). Although *G. lucidum* polysaccharide has been shown to directly kill cancer cells and activate immune cells *in vitro* (23, 24), recent research has shown that the anticancer function of *G. lucidum* polysaccharide is partially mediated by modulating the gut microbiota *in vivo* (25, 26). Whether *G. lucidum* polysaccharide exerts protective effects on cachexia and gut microbiota dysbiosis induced by the combination cisplatin plus docetaxel (cisplatin + docetaxel) remains unclear. For testing whether *G. lucidum* polysaccharide can alleviate chemotherapy-induced muscle wasting through proteolysis inhibition in muscle tissue or regulation of microbial community composition, the effects of *G. lucidum* polysaccharide on body weight, food intake, muscle wasting, and microbiota composition in cisplatin + docetaxel-treated mice were investigated. The results revealed that oral administration of *G. lucidum* polysaccharide alleviated cisplatin + docetaxel-induced cachexia (body weight loss and muscle atrophy) and other side effects, such as neutropenia. *G. lucidum* polysaccharide restored cisplatin + docetaxel-induced muscle atrophy by inhibiting the ubiquitin-proteasome pathway via reduction in the expression of *MuRF-1* and *Atrogin-1* in muscle tissue.

## RESULTS

### Effects of Liz-H on body weight and food and water consumption in cisplatin + docetaxel-treated mice.

**FIG 1:**
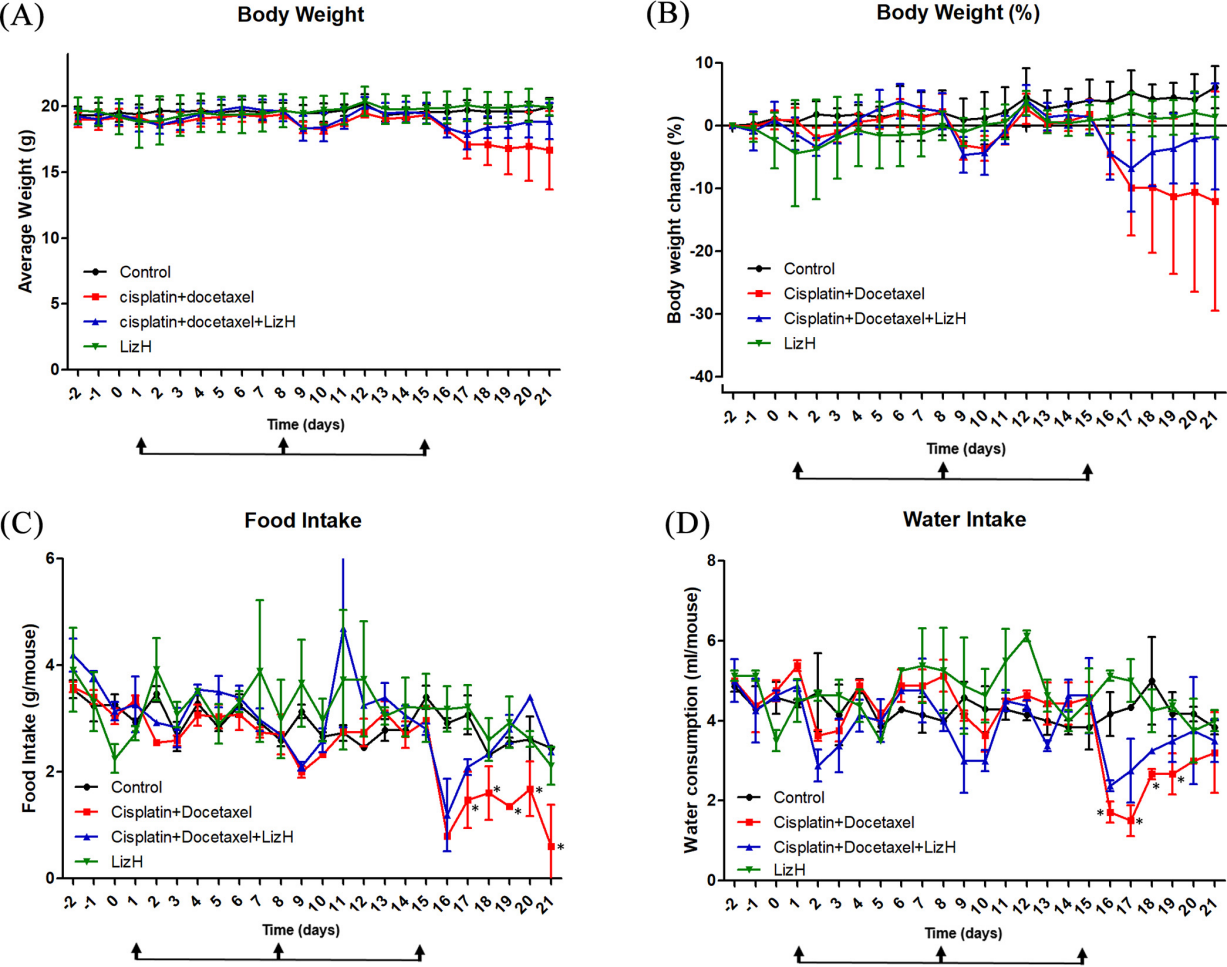
Effects of Liz-H on body weight and food and water consumption in cisplatin + docetaxel-treated mice. Body weight (A), body weight change (B), food intake (C), and water intake (D) were measured daily. Data are presented as means ± SD for the control (*n* = 7), cisplatin + docetaxel (*n* = 6), cisplatin + docetaxel + Liz-H (*n* = 8), and Liz-H (*n* = 8) groups. The asterisk indicates statistical significance compared with the docetaxel + cisplatin + Liz-H group (*P* < 0.05).

A previous study indicated that combined treatment of cisplatin and docetaxel causes body weight loss in mice (27). Whether Liz-H has a protect effect on cisplatin + docetaxel-induced weight loss was investigated. At day 21, the body weight of the cisplatin + docetaxel group was reduced compared with that of the control group (Fig. 1A and B), although not significantly. Combined treatment with Liz-H alleviated cisplatin + docetaxel-induced body weight loss (Fig. 1A and B). Anorexia is also a factor for chemotherapy-induced malnutrition, and cisplatin + docetaxel-induced weight loss may be caused by reduction in food and water consumption. The amount of food and water consumption was measured in the period of treatment daily. Similar to the body weight results, the food and water consumption was reduced at cisplatin + docetaxel injection time points, days 1, 8, and 15, compared with that in the control group (Fig. 1C and D). Combined treatment with Liz-H could restore cisplatin + docetaxel-reduced food and water consumption (Fig. 1C and D). These results indicated that the protective effect of Liz-H in chemotherapy-induced body weight loss may be mediated by stimulating appetite.

### Reduced neutropenia in cisplatin + docetaxel-treated mice by Liz-H.

Cisplatin and docetaxel are also known to generate neutropenia in patients with cancer (28), which can lead to low immune function and delay the course of chemotherapy. To test whether Liz-H could ameliorate chemotherapy-induced neutropenia, we collected blood samples from mice at the time of sacrifice. The complete blood count (CBC) results showed that white blood cells (WBCs), platelets, and lymphocytes were reduced in the cisplatin + docetaxel group and that this reduction was restored by Liz-H treatment (Table 1). This result indicated that Liz-H has a protective effect on cisplatin + docetaxel-induced neutropenia. Despite reports of chemotherapy causing kidney and liver toxicity, our findings indicated that the concentration of aspartate transaminase (AST) and alanine aminotransferase (ALT), markers of liver damage, did not increase in the group receiving cisplatin + docetaxel (Table 2). We also found that the blood glucose of cisplatin + docetaxel group mice was lower than that of control mice, and Liz-H treatment restored the blood glucose concentration (Table 2). It is possible that this protective effect was mediated through the increased food intake in the cisplatin + docetaxel + Liz-H-treated mice (Fig. 1C).

**TABLE 1:** Effects of Liz-H on complete blood count in chemotherapy reagent-treated mice^*a*^

Parameter	Value for group		
	Control	Cis + Doc	Cis + Doc + Liz-H
WBC (×10^3^/μL)	9.7 ± 0.4	4.4 ± 3.5*	7.4 ± 1.0
RBC (×10^6^/μL)	9.6 ± 0.3	7.3 ± 1.1*	7.8 ± 1.2
HGB (g/dL)	14.3 ± 0.3	11.3 ± 2.0*	12.5 ± 0.4
HCT (%)	50.2 ± 1.1	38.5 ± 5.2*	41.9 ± 7.9
MCV (fL)	52.5 ± 0.7	52.7 ± 1.1	53.6 ± 2.3
MCH (pg)	14.9 ± 0.4	15.4 ± 0.5	13.9 ± 6.4
MCHC (g/dL)	28.4 ± 0.5	29.2 ± 1.5	30.8 ± 6.5
PLT (×10^3^/μL)	1,111 ± 219.8	680.3 ± 376.5	1,348.3 ± 573.3
LYM (%)	87.1 ± 1.6	63.3 ± 31.5	84.9 ± 3.3
LYM (×10^3^/μL)	8.4 ± 0.4	3.6 ± 3.6*	6.3 ± 0.8
RDW, SD (fL)	27.3 ± 0.2	33.3 ± 1.0*	32.2 ± 3.6*
RDW, CV (%)	12.8 ± 0.7	18.7 ± 0.8	17.1 ± 2.0
MPV (fL)	6.1 ± 0.2	6.6 ± 0.8	6.7 ± 0.5

a The asterisks indicate significant differences compared with the control group (*P* < 0.05) analyzed by one-way ANOVA. Data are presented as means ± SD (*n* = 4). Cis, cisplatin; Doc, docetaxel; WBC, white blood cells; RBC, red blood cells; HGB, hemoglobin; HCT, hematocrit; MCV, mean cell volume; MCH, mean corpuscular hemoglobin content; MCHC, mean corpuscular hemoglobin concentration; PLT, platelets; LYM, lymphocytes; RDW, red cell distribution width; CV, coefficient of variation; MPV, mean platelet volume.

**TABLE 2:** Effects of Liz-H on blood biochemistry in chemotherapy reagent-treated mice^*a*^

Parameter	Value for group		
	Control	Cis + Doc	Cis + Doc + Liz-H
Glucose (mg/dL)	317.3 ± 50.2	186.5 ± 84.6*	264.3 ± 65.9
Total cholesterol (mg/dL)	65.5 ± 2.1	82.3 ± 22	83.5 ± 9.2
Blood urea nitrogen (mg/dL)	30 ± 2.2	25.5 ± 9	28.8 ± 7
T-bilirubin (mg/dL)	0.65 ± 0.48	0.325 ± 0.19	0.3 ± 0.14
GPT/ALT (1 U/L)	48.3 ± 17.7	404.5 ± 455.6	46.8 ± 20.4
GOT/AST (1 U/L)	34.3 ± 18.8	90.5 ± 93.7	16.8 ± 6.2

a The asterisks indicate significant differences compared with the control group (*P* < 0.05) analyzed by one-way ANOVA. Data are presented as means ± SD (*n* = 4 mice/group). GPT, glutamic pyruvic transaminase; GOT, glutamic oxalacetic transaminase.

### Reduction of cisplatin + docetaxel-induced muscle atrophy by Liz-H.

**FIG 2:**
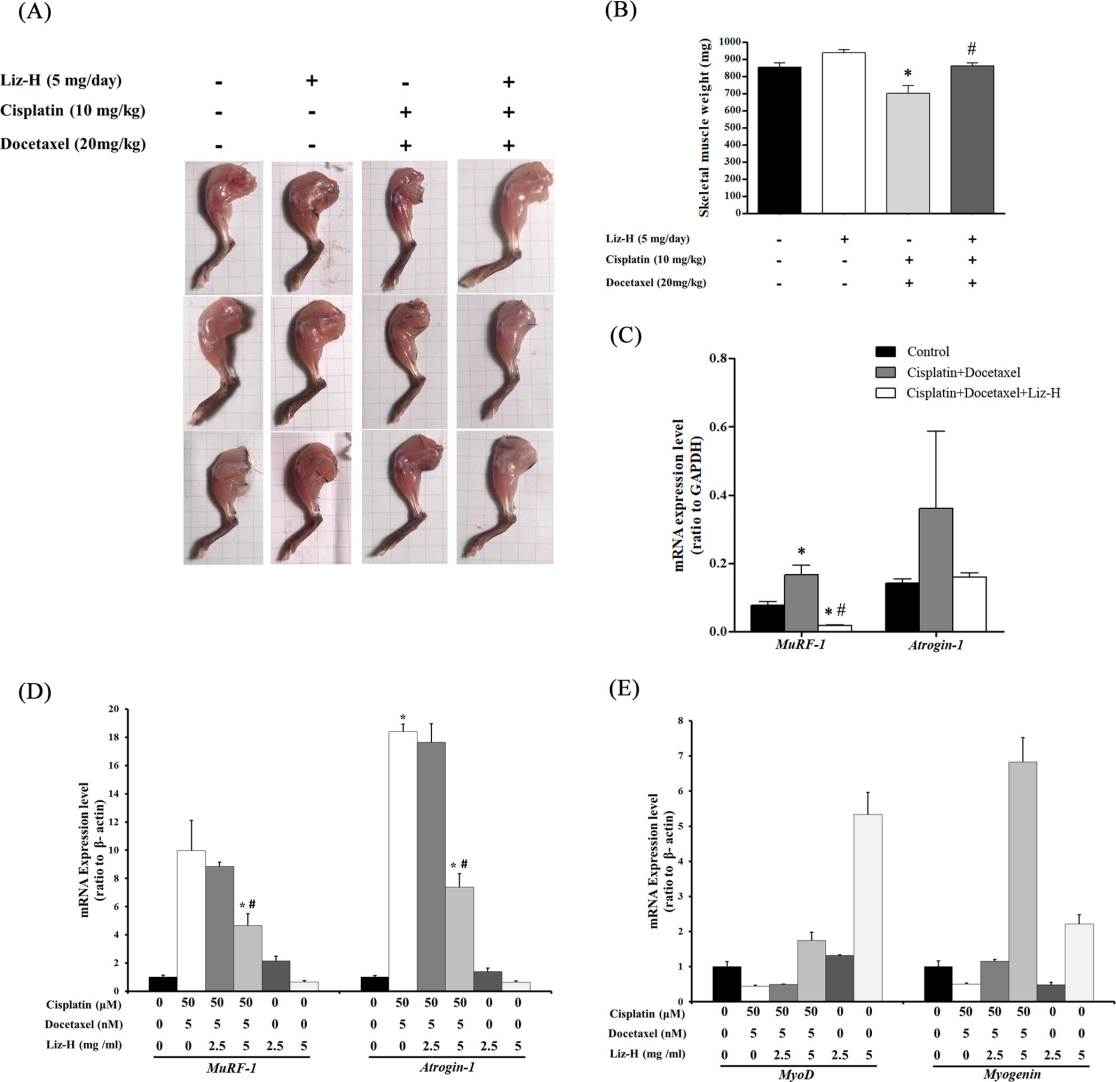
Reduction in cisplatin + docetaxel-induced muscle atrophy by Liz-H. (A to C) Macroscopic observation (A), measured weight (B), and *MuRF-1* and *Atrogin-1* mRNA levels (C) of mouse gastrocnemius muscles in the indicated groups on the day of sacrifice. (D and E) C2C12 myotubes were treated with indicated conditions for 24 h and the mRNA levels of *MuRF-1* and *Atrogin-1* (D) and MyoD and myogenin (E) were determined by qPCR. Values are presented as means ± SD. The asterisk indicates statistical significance compared with the control group (*P* < 0.05). The pound sign indicates statistical significance compared with the cisplatin + docetaxel group (*P* < 0.05). GAPDH, glyceraldehyde-3-phosphate dehydrogenase.

Liz-H did not significantly restore overall weight loss (Fig. 1), but it restored muscle mass, and the most important hallmark of cachexia is muscle loss. At day 21, the size and weight of gastrocnemius muscles in cisplatin + docetaxel-treated mice were significantly reduced compared with those in the control group (Fig. 2A and B). As expected, Liz-H reversed the loss of muscle size and weight in cisplatin + docetaxel-treated mice. These results shows that Liz-H affects cachexia and does not just cause water and fat weight gain through increasing appetite. Next, we investigated whether the mechanism of Liz-H inhibits muscle atrophy induced by cisplatin and docetaxel. The proteolysis genes *MuRF-1* and *Atrogin-1* were measured by reverse transcription-quantitative PCR (RT-qPCR). The results showed that when mice were treated with cisplatin + docetaxel, the expression levels of *MuRF-1* and *Atrogin-1* increased; however, they were significantly reduced with Liz-H combined treatment (Fig. 2C). This phenomenon was further confirmed *in vitro* with C2C12 myoblast-derived myotubes. When cultured myotubes were treated with cisplatin + docetaxel, the expression levels of *MuRF-1* and *Atrogin-1* increased and those of myogenic transcription factors MyoD and myogenin decreased (Fig. 2D and E). Liz-H treatment could alleviate the activation of *MuRF-1* and *Atrogin-1* and the reduction in MyoD and myogenin (Fig. 2D). These results indicated that Liz-H could inhibit cisplatin + docetaxel-induced proteolysis and maintain healthy expression of myogenic transcription factors.

### Effect of Liz-H on cisplatin + docetaxel-induced dysbiosis in the intestinal microbiome.

**FIG 3:**
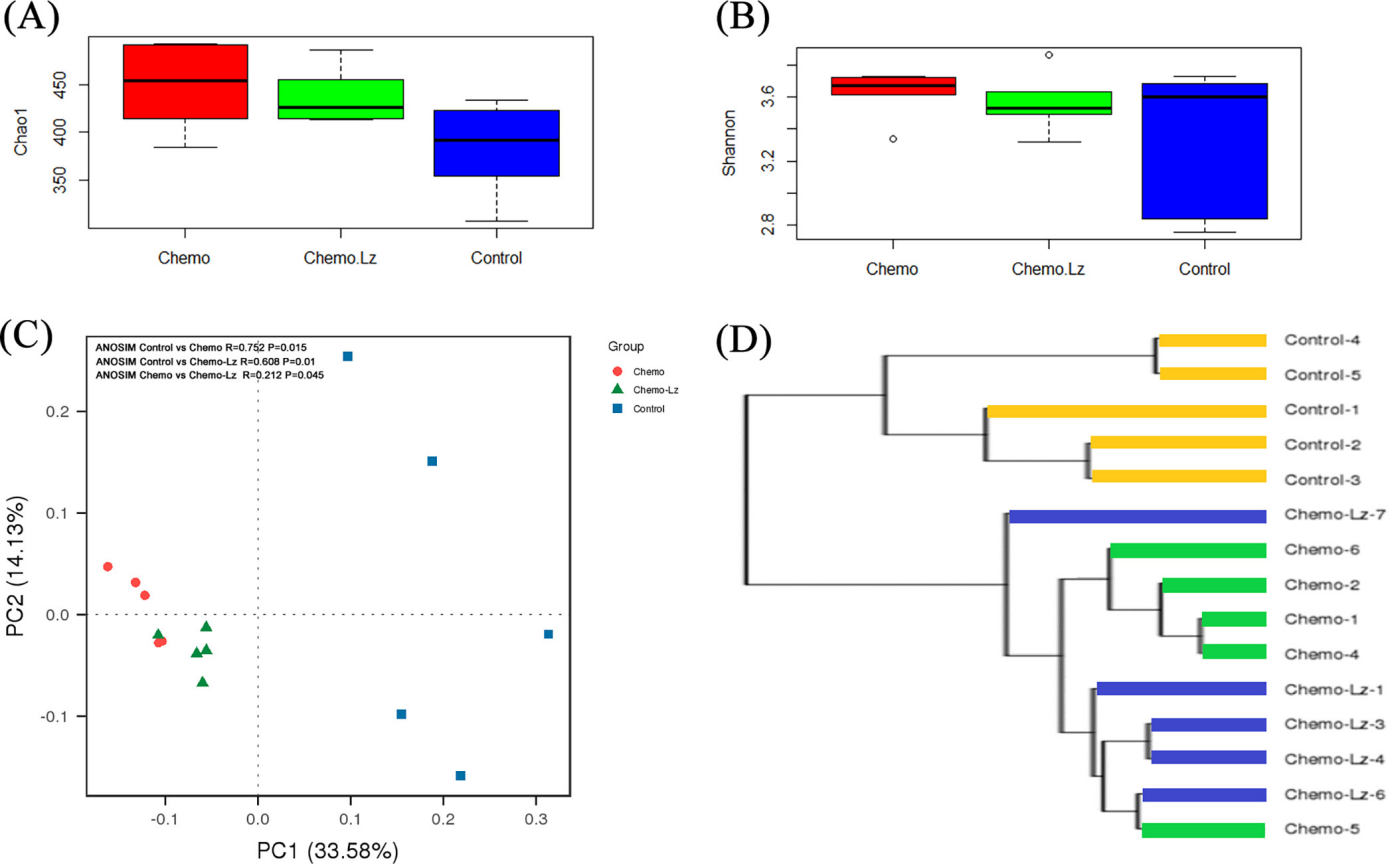
Effect of Liz-H on the diversity of gut microbiota. Alpha diversity, Chao1 index (A) and Shannon index (B). Beta diversity, principal-coordinate analysis of weighted UniFrac distances (C) and UPGMA cluster tree analysis (D).

**FIG 4:**
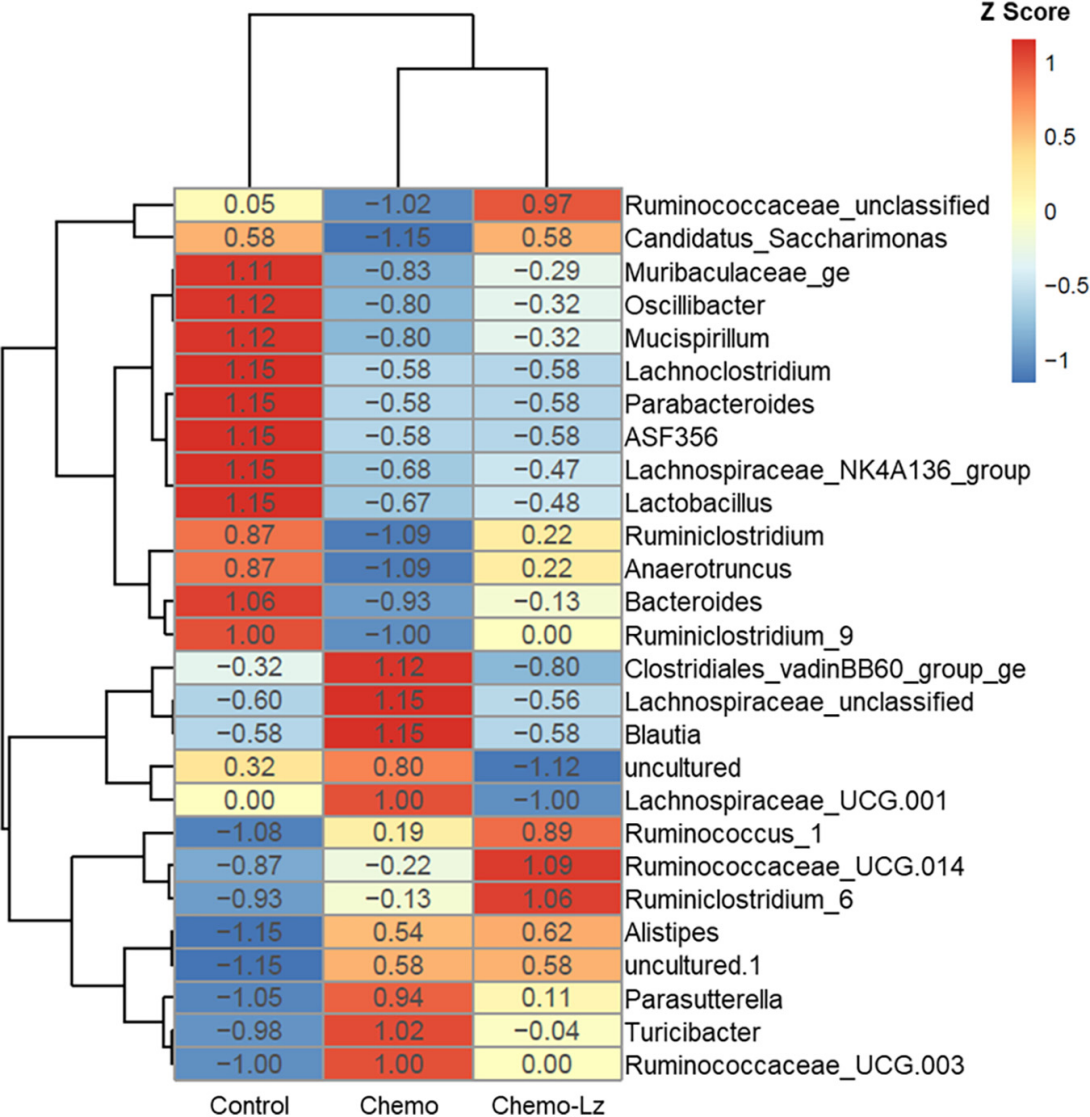
Response of gut microbiota at the genus level to Liz-H treatment. Shown is a heat map of hierarchical clustering of bacterial microbiota composition profiles represented by 16S rRNA amplicons of different indicated groups (*n* = 5 for each group). RNA from cecal contents was isolated at 21 days after the first cisplatin and docetaxel injection. The *x* axis represents the distribution of bacterial communities in the sampled depths, and the *y* axis represents the class-level taxonomy. The heat map is color-coded on the basis of Z-scores.

*G. lucidum* polysaccharide has been shown to modulate the gut microbiota in some animal models (29–35). To investigate whether Liz-H could modulate the gut microbiota composition in cisplatin + docetaxel-treated mice, 16S rRNA gene sequencing was performed to identify the composition of the gut microbiota. Alpha diversity, shown by Chao-1 and Shannon indices, was used to represent in-group microbial community diversities, with a higher index level indicating higher diversity. The results showed that the Chao-1 and Shannon indices of the cisplatin + docetaxel group were higher than those of the control group and the cisplatin + docetaxel + Liz-H group (Fig. 3A and B). In principal-coordinate analysis (PCoA), the cisplatin + docetaxel + Liz-H group was closer to the cisplatin + docetaxel group than to the control group (Fig. 3C). These results suggest that Liz-H does not globally restore the gut microbiota to control levels. UPGMA (unweighted pair group method using average linkages) cluster tree analysis also indicated that the relationship of the cisplatin + docetaxel group and the cisplatin + docetaxel + Liz-H group was more similar than the control group (Fig. 3D). However, Liz-H did seem to have an effect on distinct microbial populations in the gut. According to species annotation, *Ruminococcaceae*, “*Candidatus* Saccharimonas,” *Ruminiclostridium*, *Anaerotruncus*, *Bacteroides*, and *Ruminiclostridium* 9 were reduced in the cisplatin + docetaxel group and restored to normal levels in the cisplatin + docetaxel + Liz-H group compared with the control group (Fig. 4). *Ruminococcaceae* and *Bacteroides* have been reported to be associated with cachexia generation (36–38). The findings suggest that Liz-H may modulate these bacteria to alleviate chemotherapy-induced cachexia.

### Liz-H augmentation of cisplatin and/or docetaxel cytotoxicity in LLC cells.

**FIG 5:**
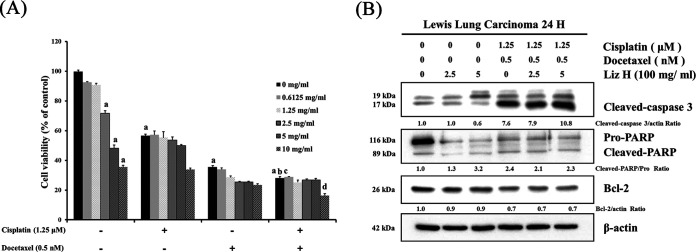
Effects of Liz-H on cell survival and apoptosis-related protein expression in LLC cells after treatment with cisplatin and docetaxel. LLC cells were treated as indicated for 24 h. Cell viability was measured by MTT assay (A), and expression of apoptosis-related protein was determined by Western blotting (B). The letter a indicates statistical significance compared with the group treated with 0 μM cisplatin + 0 nM docetaxel + 0 mg/mL of Liz-H (*P* < 0.05). The letter b indicates statistical significance compared with the group treated with 1.25 μM cisplatin + 0 nM docetaxel + 0 mg/mL of Liz-H (*P* < 0.05). The letter c indicates statistical significance compared with the group treated with 0 μM cisplatin + 0.5 nM docetaxel + 0 mg/mL of Liz-H (*P* < 0.05). The letter d indicates statistical significance compared with the group treated with 1.25 μM cisplatin + 0.5 nM docetaxel + 0 mg/mL of Liz-H (*P* < 0.05).

In this study, Liz-H reduced cisplatin + docetaxel-induced cachexia (weight loss and muscle atrophy) and neutropenia. However, it remained unclear whether the protective effect of Liz-H stemmed from suppressing the cytotoxic effects of cisplatin + docetaxel. 3-(4,5-Dimethyl-2-thiazolyl)-2,5-diphenyl-2H-tetrazolium bromide (MTT) assay was performed to confirm the survival rate of Lewis lung carcinoma (LLC) cells treated with different concentrations of Liz-H with or without cisplatin + docetaxel. The results indicated that when LLC cells were treated with Liz-H alone, the survival rate was reduced in a dose-dependent manner. Moreover, Liz-H coadministered with cisplatin and/or docetaxel could enhance the cytotoxicity (Fig. 5A). The levels of cleaved caspase 3 and anti-apoptotic protein Bcl-2 are indicative of cellular apoptosis. We found that the cleaved caspase 3 slightly increased in Liz-H cotreatment with cisplatin and docetaxel in a dose-dependent manner. Bcl-2 expression was slightly reduced by Liz-H cotreatment with cisplatin and docetaxel (Fig. 5B). These results indicated that Liz-H treatment dose not suppress the anticancer effects of cisplatin + docetaxel and may in fact enhance them.

## DISCUSSION

Side effects from cancer chemotherapy cause discomfort in patients, delay the therapeutic course, and can even cause death. Cisplatin and docetaxel combination therapy is mainly used in non-small cell lung cancer. Relatively few patients are treated with surgery because the cancer is usually found after metastasis has occurred. In this study, treatment with *G. lucidum* polysaccharide Liz-H could reduce cisplatin + docetaxel-induced cachexia (weight loss and muscle atrophy) and neutropenia, indicating that Liz-H is a good chemoprotective agent. Furthermore, when combined with Liz-H, chemotherapeutic drugs retained their cancer-killing function *in vitro* (Fig. 5). Similar results were demonstrated by Qiu et al., who found that *G. lucidum* polysaccharide combined with cisplatin could enhance the inhibition of lung cancer (23). Although this study mainly focused on the effect of *G. lucidum* polysaccharides, triterpenoids and fungal immunomodulatory proteins (FIPs) are also biologically active in *G. lucidum* extracts. Both of them also act as chemoprotective agents. The triterpenoids of *G. lucidum* have been shown to prevent cisplatin-induced nephrotoxicity (39). Our group’s previous study indicated that FIP-gts could alleviate docetaxel-induced myelosuppression and intestinal mucosal damage (40). Another *G. lucidum* FIP, LZ-8, ameliorated cyclophosphamide-induced leukopenia (41). The potential use of nanomaterials as elicitors to increase the production of valuable metabolites by medicinal fungi has received attention. More recently, it has been revealed that graphene-based nanomaterials can activate the production of ganoderic acid (42).

Cachexia is a multifactorial syndrome that causes extreme weight loss and muscle atrophy. Evidence indicates that the muscle atrophy induced by cachexia is mediated by activating E3 ubiquitin ligases, MuRF-1 and atrogin-1, and results in protein degradation in muscle (43). This study was the first to report that *G. lucidum* polysaccharides could alleviate chemotherapy-induced cachexia via reducing *MuRF-1* and *Atrogin-1* expression. As shown in Fig. 5B, in C2C12 cells treated with *G. lucidum* polysaccharides alone, the expression of myogenic factors MyoD and myogenin increased. This result indicated that *G. lucidum* polysaccharides also play a role in muscle differentiation.

Up to this point, no direct evidence has been presented to demonstrate that *G. lucidum* polysaccharide can be absorbed by the intestine. Given that there are no suitable polysaccharide-digesting enzymes generated by the human gut, we speculate that *G. lucidum* polysaccharide cannot be absorbed directly by the human intestine. Instead, we suggest that the polysaccharide may be decomposed by the gut microbiota and subsequently inhibit chemotherapy-induced cachexia. In addition, our findings in Fig. 2D and E indicate that the polysaccharide can also directly affect the expression of *MuRF-1*, *Atrogin-1*, *MyoD*, or *Myogenin*, suggesting that it may work *in vivo* through both direct and indirect mechanisms. To bypass any potential effects of Liz-H metabolism by the microbiota, it may be advisable to consider conducting an experiment in the future in which Liz-H is administered intravenously or intraperitoneally.

To confirm whether the effect of Liz-H is related to its influence on the intestinal microbiota, we fed mice in the cisplatin + docetaxel group with fecal samples from Liz-H mice. However, we found that fecal microbiota transplantation (FMT) did not significantly prevent the weight loss, muscle atrophy, or *MuRF-1* and *Atrogin-1* increase induced by cisplatin + docetaxel treatment (see Fig. S1 in the supplemental material). We believe that the unexpected outcome could be attributed to the inadequate presence of effective bacteria. It would be intriguing to investigate whether reintroducing *Bacteroides* and *Ruminococcaceae* into the gut of cisplatin + docetaxel-treated mice would be adequate in mitigating some of the side effects associated with chemotherapy.

Overall intestinal dysbiosis induced by cisplatin + docetaxel was not changed by Liz-H cotreatment. Regarding beta diversity (Fig. 3C), the compositions of the microbiota were similar in the cisplatin + docetaxel group and the cisplatin + docetaxel + Liz-H group and far from the control group. However, Liz-H did seem to recover levels of specific bacterial species. Based on species annotation, *Ruminococcaceae*, “*Candidatus* Saccharimonas,” *Ruminiclostridium*, *Anaerotruncus*, *Bacteroides*, and *Ruminiclostridium* 9 were reduced in the cisplatin + docetaxel group and recovered by Liz-H treatment, indicating that they may play a role in the anti-cachexia effect of Liz-H. Consistent with the present study’s findings, a reduction in the abundance of butyrate produced by *Ruminococcaceae* was also found in the cachectic mouse colon carcinoma 26 (C26) model (36, 37), indicating that *Ruminococcaceae* and their metabolite butyrate may be important in cachexia prevention. Butyrate also ameliorates skeletal muscle atrophy in a diabetic nephropathy model (44). Bindels et al. demonstrated that the abundance of *Bacteroides* was also reduced in cachectic leukemia: when the mice were treated with nondigestible pectic oligosaccharides, the amount of *Bacteroides* increased and cachexia was reduced (38). Another report also showed a decrease in *Bacteroidia* in a Toxoplasma gondii-induced cachexia model (45), suggesting that the reduction of *Bacteroides* may be a common feature of certain cachexia models. Although some bacterial changes may mediate the function of Liz-H, no direct evidence could confirm this hypothesis. Thus, further verification is needed.

In conclusion, this study indicated that Liz-H is a good chemoprotective reagent for cisplatin + docetaxel-induced cachexia, side effects, and dysbiosis.

## MATERIALS AND METHODS

### Reagents.

Docetaxel (Isotera) was purchased from Nang Kuang Pharmacecutical Co. Ltd. Cisplatin was obtained from Sigma (number P4394). *G. lucidum* polysaccharide Liz-H (Shuang Hor B Ganoderma) was produced from Double-Crane Pharmaceuticals Co. Ltd. Briefly, *G. lucidum* YK-01 (Lingzhi Agricultural Co. Ltd.) was maintained on an malt extract agar (MEA) plate at 26°C. Seed culture was performed in 400 mL of seed medium (60 g/L of glucose, 15 g/L of sucrose, 1.8% yeast extract, 0.3% yeast peptone, and 0.6 g/L of KH_2_PO_4_) at 28°C for 10 days. Then, 3 L of seed culture was transferred into a 50-L fermentor and cultured in fermentation medium (70 g/L of glucose, 3.2 g/L of yeast powder, 2 g/L of yeast peptone, 1.5 g/L of MgSO_4_, and 0.06 g/L of KH_2_PO_4_). The whole culture was harvested and freeze-dried when the glucose concentration was below 1% in the fermentation medium.

### Cell culture.

The Lewis lung carcinoma (LLC) cell line was maintained in Dulbecco’s modified Eagle’s medium (DMEM) supplemented with 10% fetal bovine serum, 2 mM l-glutamine, and 100 U/mL of penicillin-streptomycin at 37°C in an atmosphere of 95% air and 5% CO_2_. C2C12 myoblasts were maintained in DMEM supplemented with 10% fetal bovine serum (HyClone), 2% HEPES, 2 mM l-glutamine, and 100 U/mL of penicillin-streptomycin at 37°C in an atmosphere of 95% air and 5% CO_2_. Fusion and differentiation of C2C12 cells were performed using the following procedure. A total of 8 × 10^4^ cells were seeded in a 6-cm culture dish and cultured for 48 h. Then the medium was replaced with differentiation medium (DMEM supplemented with 2% horse serum and 100 U/mL of penicillin-streptomycin) and cells were cultured for 5 days. When 90% of cells were differentiated into myocytes, they were treated with cisplatin + docetaxel in the presence or absence of Liz-H. The viability of LLC cells was measured by MTT assay. Briefly, following treatment with Liz-H, cells were further incubated with 0.5 mg/mL of MTT in culture medium for 3 h. Then the formazan was dissolved with dimethyl sulfoxide and absorbance at a wavelength of 570 nm was measured.

### Animals.

Female C57BL/6J mice (6 to 8 weeks old) weighing 19 to 22 g were purchased from the National Laboratory Animal Center (Taipei, Taiwan). All animal studies were conducted in accordance with the protocols approved by the Institutional Animal Care and Use Committee (IACUC) of Chung Shan Medical University (IACUC approval number 1834-2207-1728).

### Animal study design.

**FIG 6:**
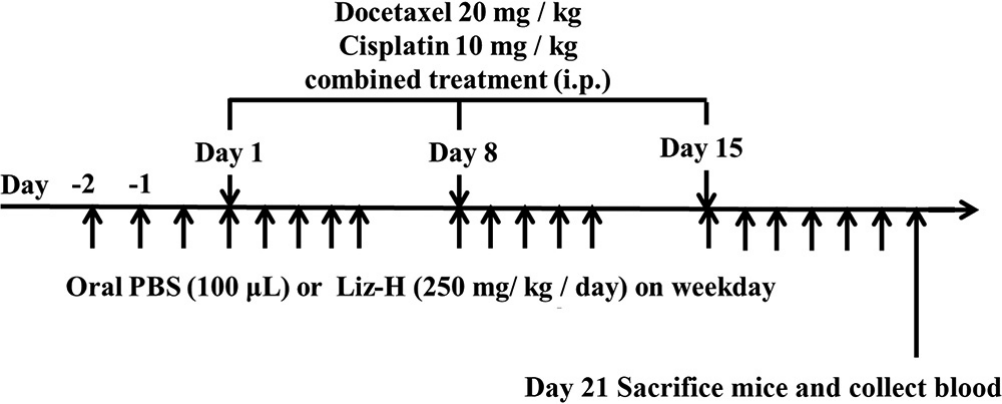
Schematic of experimental procedure. C57BL/6J mice (6 to 8 weeks old) were divided into four groups and treated with the following: (i) PBS as a control, (ii) docetaxel + cisplatin, (iii) docetaxel + cisplatin + Liz-H, and (iv) Liz-H. Docetaxel + cisplatin-injected mice were pretreated with PBS or Liz-H (5 mg/day) for 3 days before docetaxel injection. Docetaxel + cisplatin was injected on days 1, 8, and 15. At day 11, blood samples were collected, and on day 21, the mice were sacrificed.

Following 1 week of acclimatization, the mice were randomly divided into four groups: (i) the control group, in which from day 2, 100 μL of phosphate-buffered saline (PBS) was administered by gavage on weekdays for 3 weeks; (ii) the cisplatin + docetaxel group, in which cisplatin (10 mg/kg of body weight) and docetaxel (20 mg/kg) were administered by intraperitoneal (i.p.) injection on days 1, 8, and 15 and 100 μL of PBS was administered by gavage on weekdays for 3 weeks; (iii) the cisplatin + docetaxel + Liz-H group, in which from day 2, Liz-H (250 mg/mouse) was administered by gavage on weekdays for 3 weeks and cisplatin + docetaxel was administered by i.p. injection on days 1, 8, and 15; and (iv) the Liz-H group, in which Liz-H (250 mg/mouse) was administered by gavage on weekdays for 3 weeks. A schematic of the treatment is shown in Fig. 6. Body weight and food and water consumption were measured before gavage daily. All animals were sacrificed by CO_2_ euthanasia on day 21. Blood samples were obtained from the inferior vena cava, and complete blood count (CBC) and blood biochemistry were analyzed by Hemavet automated cell counter and reagents from Denka Seiken Co. Ltd., respectively. The gastrocnemius muscles were immediately removed, weighed, and then snap-frozen in liquid nitrogen prior to storage at −80°C.

### Gut microbiota analysis.

The contents of the cecum were obtained on day 21 and the total genomic DNA was isolated using Qiagen QIAamp fast DNA stool minikit in accordance with the manufacturer’s instructions. The V3-V4 region of the 16S rRNA gene was amplified by PCR. The amplicon library was sequenced using the Illumina Solexa platform at Welgene Biotech Co. Ltd., Taipei, Taiwan. After sequencing, the operational taxonomic units (OTUs) were determined by the sequence similarity and used for the following analysis. The alpha (Chao1 index and Shannon index) and beta diversities (PCoA and UPGMA) of different groups were calculated using Qiime software. The heat map of hierarchical clustering of bacterial microbiota composition was created using RStudio software.

### Western blotting.

Anti-poly(ADP-ribose) polymerase (anti-PARP; number 9542; Cell Signaling, Danvers, MA, USA), anti-cleaved caspase-3 (number 9661; Cell Signaling), anti-Bcl-2 (number 2870; Cell Signaling), anti-β-actin (Sigma; A5441), anti-mouse IgG-horseradish peroxidase (HRP) (number 7076; Cell Signaling), and anti-rabbit IgG-HRP (number 7074; Cell Signaling) were used to detect the expression levels of the indicated proteins. The complete protocol for Western blot assay has been described in a previous publication (46).

### RNA extraction and quantitative real-time PCR analysis.

Isolated gastrocnemius muscles were immersed in liquid nitrogen and then ground into powder for further RNA extraction. All RNA extractions were performed using a rare RNA reagent (Genepure Technology) in accordance with the manufacturer’s instructions. Briefly, 3 μg of total RNA was used to perform reverse transcription using a high-capacity cDNA reverse transcription kit (Applied Biosystems; 4368813). Real-time PCR was carried out with primers (Table S1) and Smart Quant green master mix (Protech; PT-GL-SQGLR-V3) in an ABI StepOnePlus real-time PCR system.

### Statistical analysis.

A one-sample *t* test by IBM SPSS Statistics 19 was used for all statistical analyses, except the CBC and blood biochemistry data, which were analyzed by one-way analysis of variance (ANOVA). Data are presented as means ± standard deviations (SD). *P* values of <0.05 were considered significant. The statistical analysis of PCoA was performed by analysis of similarity (ANOSIM) with RStudio software.

### Data availability.

All raw sequencing data have been submitted to the NCBI Sequence Read Archive (SRA) database under accession numbers SRR23501582 to SRR23501596.
