# Supplementary material for: Polysaccharide of Ganoderma lucidum Ameliorates Cachectic Myopathy Induced by the Combination Cisplatin plus Docetaxel in Mice
Source: Microbiol Spectr. 2023 May 22;11(3):e03130-22. doi: 10.1128/spectrum.03130-22 (PMC10269453; doi:10.1128/spectrum.03130-22)
Supplement: Supplemental file 1 — Supplemental material. Download spectrum.03130-22-s0001.pdf, PDF file, 0.5 MB [file spectrum.03130-22-s0001.pdf]

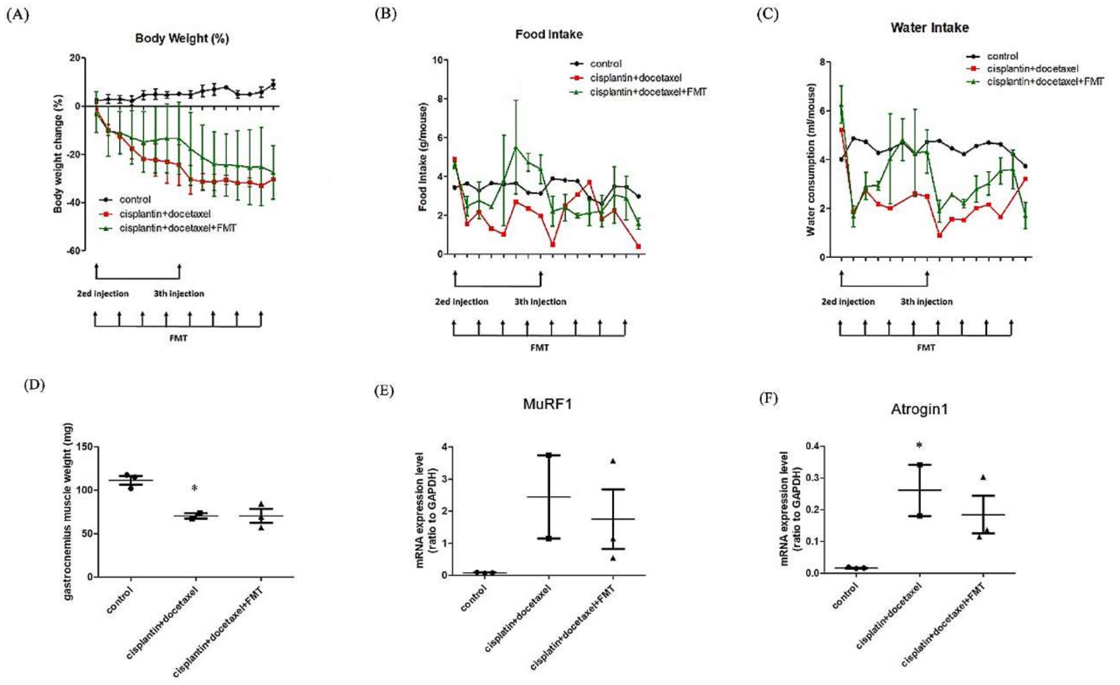

Figure S1. FMT for chemotherapy mice. Female-C57BL/6J mice (6–8 weeks old) were divided into three groups (n=3, each group) and treated as follows:(1) control (2) cisplatin + docetaxel (3) cisplatin + docetaxel + FMT. In cisplatin + docetaxel and cisplatin + docetaxel + FMT groups, each mouse was injected with 0.4 mg Docetaxel and 0.2 mg cisplatin at day1, but half dosage at day 14 and day 21. The fresh isolated faeces from Liz-H treated mice (already feed Liz-H for 2 weeks) were suspended in PBS at concentration 100 mg/ml, and then treat cisplatin + docetaxel + FMT mice with 100 ul per mice by gavage every 2 days. The body weight change (%) (A), Food intake (B), and Water intake (C) were measured every day. All mice were sacrificed at day 29, and the weight of gastrocnemius muscle were measured (D). The expression level of MuRF-1 (E) and Atrogin-1 (F) is measured by real time PCR.

TABLE S1 Primer Sequence	
mice Myogenin	F:5′-ACAGCATCACGGTGGAGGATATGT-3′<br>R:5′-CCCTGCTACAGAAGTGATGGCTTT-3′
mice MyoD	F:5′-ACGACTGCTTTCTTCACCACTCCT-3′<br>R:5′-TCGTCTTAACTTTCTGCCACTCCG-3′
mice MuRF1	F: 5′-CTGGTGGCTATTCTCCTTGG-3′<br>R: 5′-GGTGCCTACTTGCTCCTTGT-3′
mice Atrogin-1	F: 5′-CGACCTGCCTGTGTGCTTAC-3′<br>R: 5′-CTTGCGAATCTGCCTCTCTG-3′
mice β-actin	F: 5′-CAACCTTCTTGCAGCTCCTC-3′<br>R: 5′-TTCTGACCCATACCCACCAT-3′
mice GAPDH	F: 5′-CCAGCCTCGTCCCGTAGAC-3′<br>R: 5′-CGCCCAATACGGCCAAA-3′
